# Large-scale production of magnetosomes by chemostat culture of *Magnetospirillum gryphiswaldense *at high cell density

**DOI:** 10.1186/1475-2859-9-99

**Published:** 2010-12-12

**Authors:** Yang Liu, Guo R Li, Fang F Guo, Wei Jiang, Ying Li, Lun J Li

**Affiliations:** 1 State Key Laboratories for Agro-biotechnology and College of Biological Sciences, China Agricultural University, Beijing 100193, China; 2 China National Center for Biotechnology Development, Beijing 100036, China

## Abstract

**Background:**

Magnetotactic bacteria have long intrigued researchers because they synthesize intracellular nano-scale (40-100 nm) magnetic particles composed of Fe_3_O_4_, termed magnetosomes. Current research focuses on the molecular mechanisms of bacterial magnetosome formation and its practical applications in biotechnology and medicine. Practical applications of magnetosomes are based on their ferrimagnetism, nanoscale size, narrow size distribution, dispersal ability, and membrane-bound structure. However, the applications of magnetosomes have not yet been developed commercially, mainly because magnetotactic bacteria are difficult to cultivate and consistent, high yields of magnetosomes have not yet been achieved.

**Results:**

We report a chemostat culture technique based on pH-stat feeding that yields a high cell density of *Magnetospirillum gryphiswaldense *strain MSR-1 in an auto-fermentor. In a large-scale fermentor, the magnetosome yield was significantly increased by adjusting the stirring rate and airflow which regulates the level of dissolved oxygen (DO). Low concentration of sodium lactate (2.3 mmol l^-1^) in the culture medium resulted in more rapid cell growth and higher magnetosome yield than high concentration of lactate (20 mmol l^-1^). The optical density of *M. gryphiswaldense *cells reached 12 OD_565 nm _after 36 hr culture in a 42 L fermentor. Magnetosome yield and productivity were 83.23 ± 5.36 mg l^-1 ^(dry weight) and 55.49 mg l^-1 ^day^-1^, respectively, which were 1.99 and 3.32 times higher than the corresponding values in our previous study.

**Conclusions:**

Compared to previously reported methods, our culture technique with the MSR-1 strain significantly increased cell density, cell yield, and magnetosome yield in a shorter time window and thus reduced the cost of production. The cell density and magnetosome yield reported here are the highest so far achieved with a magnetotactic bacteria. Refinement of this technique will enable further increase of cell density and magnetosome yield.

## Background

Magnetotactic bacteria, first described by Richard Blakemore [1], have long intrigued researchers because they synthesize intracellular nano-scale (40-100 nm) magnetic particles composed of Fe_3_O_4_, termed magnetosomes. The extensively studied strains of magnetotactic bacteria include *Magnetospirillum gryphiswaldense *MSR-1, *M. magnetotacticum *MS-1, *M. magneticum *AMB-1, *Magnetococcus sp*. MC-1, and magneto-ovoid strain MO-1 [2-6]. Interestingly, a variety of higher organisms, including bees [7], algae [8], pigeons [9], eels [10], and humans [11], are also capable of synthesizing intracellular magnetite. The formation and physiological function of magnetic crystals in these organisms are not known. However, thorough understanding of bacterial magnetosome formation will serve as a model to uncover the mechanism of magnetosome formation and function in other species.

Current research focuses on the molecular mechanisms of bacterial magnetosome formation [12] and its practical applications in biotechnology and medicine [13]. Complete or partial genomes of *M. magnetotacticum *MS-1, *M. gryphiswaldense *MSR-1, *M. magneticum *AMB-1, *Magnetococcus sp*. MC-1 and magneto-ovoid strain MO-1 have been published [14,15]. Functional analysis of several genes involved in magnetosome formation, *e.g*., *mamJ*, *mamK*, *magA *[12,16-23] have revealed the roles of membrane associated proteins in transport and biomineralization processes required for the assembly of magnetosomes.

Practical applications of magnetosomes are based on their ferrimagnetism, nanoscale size, narrow size distribution, dispersal ability, and membrane-bound structure [13]. Bacterial magnetosomes have been used experimentally as carriers of enzymes [24], antibodies [25,26] for highly sensitive immunoassay, and as efficient sorbents for isolation and purification of DNA or RNA. Artificial magnetic nanoparticles have been used as carriers for cancer diagnosis and targeted therapy in experimental animals [27-30]. Similarly, magnetic nanoparticles enclosed in biological membranes can be linked to genes or drug molecules and thus could be used as carriers of drugs for targeted therapy of tumors [31]. Several recent reports indicate that purified, sterilized magnetosomes from *M. gryphiswaldense *MSR-1 are non-toxic for mouse fibroblasts *in vitro*, and may be useful as carriers of genes, or drugs for cancer therapy or other diseases [32,33]. However, the applications of magnetosomes have not yet been developed commercially, mainly because magnetotactic bacteria are difficult to cultivate and consistent, high yields of magnetosomes have not yet been achieved [34-37].

Recently, we described a novel culture method for high-yield growth and magnetosome production of *M. gryphiswaldense *[38], but large-scale cultivation requires further refinement of nutrient control and other culture conditions. Here we report a chemostat culture technique by pH-stat feeding, leading to rapid cell growth and maximized magnetosome formation by *Magnetospirillum gryphiswaldense *strain MSR-1 at low dissolved oxygen concentration and carbon source limitation. pH-stat feeding is a feeding strategy based on a pH feedback control. The substrate feeds into the system in response to the change in pH of the culture. This technique allows the concentrations of carbon, nitrogen, and iron sources to be easily controlled at constant levels and scaled up for large-scale preparation of magnetosomes. Moreover, it provides a useful guideline for resolving the problem of difficult cultivation of some micro-aerobic microorganisms.

## Results

### Optimal shaking conditions for flask cultures

**Figure 1:**
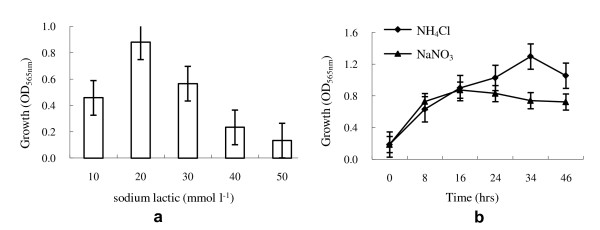
**Effects of carbon source and nitrogen source concentration on growth of *M. gryphiswaldense***. a: effect of sodium lactate concentration on cell growth. b: effects of NH_4_Cl and NaNO_3 _concentration on cell growth. All experiments are repeated three times independently and for each time three parallel samples were used to generate the average.

Experiments investigating the effects of medium components on MSR-1 cell growth in shake-flasks indicated that mineral elixir, but not vitamin elixir, is essential for culture [39]. Sodium lactate was determined to be the best carbon source (data not shown), at a maximum concentration of 20 mmol l^-1 ^(Figure 1a). NH_4_Cl was better than NaNO_3 _as the nitrogen source (Figure 1b).

### Chemostat culture

**Figure 2:**
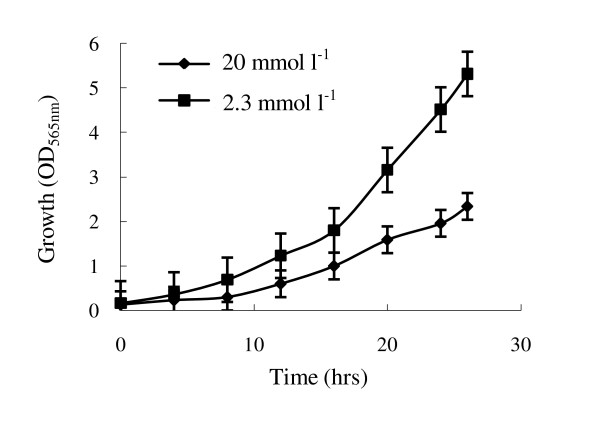
**Effect of sodium lactate concentration on cell growth of *M. gryphiswaldense *during cultivation in a 7.5 l fermentor**. The experiment was carried out in Na-lactate medium as described previously [37]. The initial Na-lactate concentrations in the medium were adjusted to 20 mmol l^-1^ and 2.3 mmol l^-1^ respectively. The concentrations of Na-lactate could be auto-controlled at a constant level during the cultivation process using the "chemostat culture" technique based on pH-stat feeding. All experiments are repeated three times independently to prove it is reproducible.

Optimized conditions for shake-flask culture are not directly applicable to large scale cultivation in fermentor. To investigate the effects of sodium lactate and NH_4_Cl concentrations on MSR-1 cell growth and magnetosome formation, we developed a "chemostat culture" technique based on pH-stat feeding during the cultivation process, to control concentrations of carbon, nitrogen, and iron sources at a constant level. The feed solution contained (per liter) 4.2 g ferric citrate, 129 g sodium lactate, 52.6 g lactic acid, and 54.9 g NH_4_Cl; these concentrations were determined in preliminary experiments. Chemostat conditions at various lactate concentrations were achieved by pH-stat feeding and adjusting initial sodium lactate in the cultivation medium. The growth rate was higher at a low concentration of sodium lactate (2.3 mmol l^-1^) than at a high concentration (20 mmol l^-1^) (Figure 2). We also studied the effects of C/N ratios 2/1, 4/1, and 6/1 on cell growth at a constant sodium lactate concentration (2.3 mmol l^-1^) in a fermentor, by regulating the concentration of NH_4_Cl. A C/N ratio of 4/1 was used in subsequent experiments as no significant influence on cell density and growth rate was observed with changes in this parameter.

### Optimal dissolved oxygen concentration (DO)

**Figure 3:**
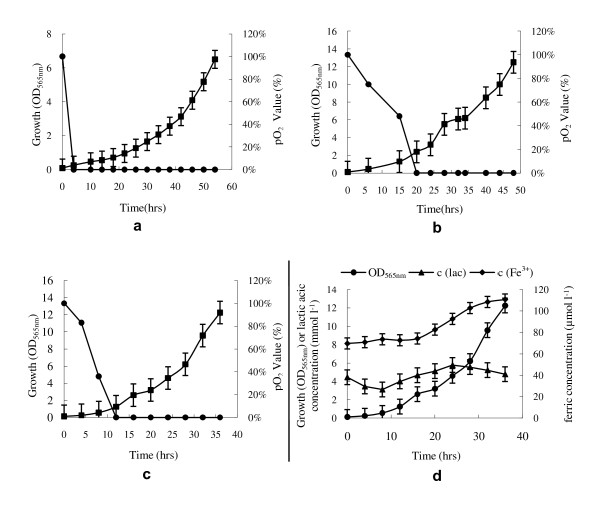
**Effect of dissolved oxygen concentration (DO) on cell growth of *M. gryphiswaldense *during large scale culture in a 42 l fermentor**. a: Cell growth and DO under initial airflow and stirring rate 0.3 l min^-1 ^and 100 r min^-1^, respectively; stirring rate was increased by 40 r min^-1 ^at 14, 20, 28, and 40 hr, successively. b: Cell growth and DO under initial airflow and stirring rate 2.0 l min^-1 ^and 200 r min^-1^; airflow was increased to 4, 8, 16 l min^-1 ^at at 24, 32, or 42 hr. c: Cell growth and DO under initial airflow and stirring rate 1.0 l min^-1 ^and 200 r min^-1^; air flow and stirring rate were adjusted to 2 l min^-1 ^at 20 hr and 300 r min^-1 ^at 28 hr, respectively. d: relationships among concentration of sodium lactate (lac), ferric citrate (Fe^3+^), and cell growth. All experiments are repeated three times independently to prove it is reproducible.

Since high density cultures yield increased levels of products, we investigated the role of oxygen in cell density in cultures. As for all magnetotactic bacteria, oxygen increased MSR-1 cell density but reduced or blocked magnetosome formation [34-36]. Thus, there is a conflict between cell growth and magnetosome formation. As shown in Figure 3 cell density of MSR-1 depended on oxygen concentration in the medium during cultivation (Figure 3a, b). This is one of the reasons why high cell density and large magnetosome yield are so difficult to achieve simultaneously with magnetotactic bacteria.

Air flow rate and stirring rate, which affect DO, were experimentally optimized for chemostat culture. In order to maintain the low DO necessary for magnetosome formation, air flow and stirring rate were initially set at 0.3 l min^-1 ^and 100 r min^-1^, respectively [38]. Under these conditions, the DO became undetectable, and the cell density was only 0.26 OD_565 nm _at 4 h of incubation (Figure 3a). In order to accelerate cell growth, the stirring rate was increased by 40 r min^-1 ^at 14, 20, 28, and 40 hr, successively; however, the DO remained at zero. Finally, the cell density reached 6.5 OD_565 nm _after 54 hr (Figure 3a). Formation of magnetosomes in the cells began one hour after the DO became undetectable, and continued along with cell growth until the end of cultivation. The dry weight and productivity of the magnetosomes were 40.0 mg l^-1 ^and 17.5 mg l^-1 ^day^-1^, respectively. Although the growth rate and magnetosome yield under these conditions were similar relative to previous results [38], it was clear that growth rate was slow at initial growth phase, resulting from the low DO. We therefore tried to enhance DO in the initial growth phase in order to accelerate growth and shorten the cultivation period.

**Figure 4:**
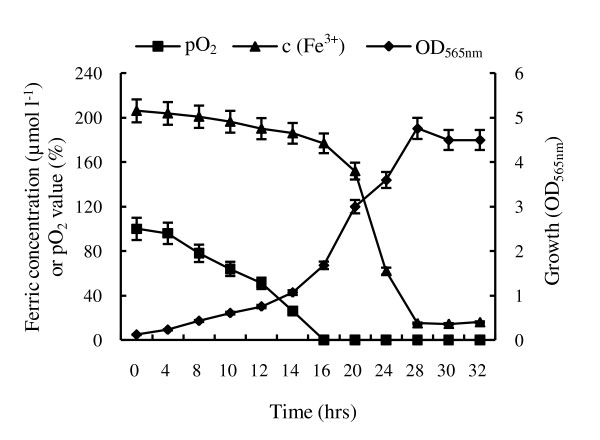
**The absorption curve of ferric ion during cultivation in a 7.5 l fermentor**. 200 μmol l^-1^was added into initial medium. All experiments are repeated three times independently to prove they are reproducible.

When the stirring rate and initial air flow were increased to 200 r min^-1 ^and 2.0 l min^-1^, respectively, the DO decreased relatively slowly and became undetectable at 20 hr whereas the growth rate increased greatly. The cell density reached 2.4 OD_565 nm _units at this point. However, similar to the foregoing results, no magnetosomes were observed by transmission electron microscopy (TEM) in this phase. Magnetosome formation occurred in all periods of growth only if DO was undetectable after 1 or 2 hr incubation (Figure 4). We subsequently increased airflow to 4, 8, or 16 l min^-1 ^at 24, 32, or 42 h, although the DO level remained undetectable. The resulting cell density and magnetosome yield were 12.5 OD_565 nm _and 60.0 mg l^-1 ^(dry weight) at 48 h, respectively (Figure 3b). The productivity was 30.0 mg l^-1 ^day^-1^. In view of these results, we increased the DO level by increasing the air flow and stirring rate during the initial growth phase, in order to enhance growth rate and magnetosome yield. However, no magnetosomes were formed in the cells until the DO level decreased to undetectable.

Due to the microaerobic character of MSR-1, initial air flow and stirring rate were maintained at 1 l min^-1 ^and 200 r min^-1 ^in order to decrease the DO during the initial growth phase of the culture for further experiments. To increase the DO in the late culture phase, air flow and stirring rate were adjusted to 2 l min^-1 ^at 20 hr and 300 r min^-1 ^at 28 hr, respectively. Under these conditions, cells grew more rapidly; DO became undetectable at 12 h, and cell density reached 12.3 OD_565 nm _within 36 h (Figure 3c). Concentrations of sodium lactate and ferric citrate were controlled between 3-6 mmol l^-1 ^and 70-110 μmol l^-1^, respectively, throughout the course of cultivation (Figure 3d). Resulting magnetosome yield and productivity were 83.23 ± 5.36 mg l^-1 ^and 55.49 mg l^-1 ^day^-1^, respectively. These values are the highest so far reported, and are 1.99 and 3.32 times higher, respectively, than those achieved in our previous study [38].

### Ferric ion uptake

**Figure 5:**
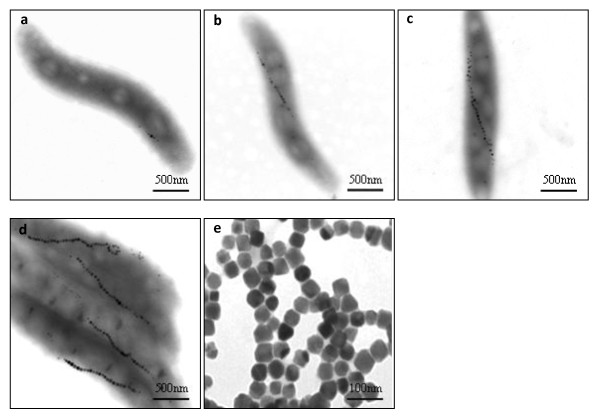
**Electron micrographs of cells in large scale culture at various phases, and purified magnetosomes**. a, b, c, d,: 1-2 hr, 3-4 hr, 15-16 hr, and 24 hr, respectively, after DO became undetectable. e: purified magnetosomes. For each sample more than 20 micrographs were got and one representative image was selected.

Since Fe_3_O_4 _is the major component of magnetosomes, we investigated the effect of ferric citrate concentration in the medium on the growth of MSR-1 and magnetosome formation in shake-flasks and fermentor. No significant effect was observed on cell growth rate in the concentration range of 20-500 μmol l^-1^. Rapid ferric ion uptake occurred in the log phase of cell growth, but not in the lag phase or the stationary phase, as shown by the absorption rate during large scale culture in fermentor (Figure 4). This result suggests that dynamic cell growth is necessary for uptake of ferric ion and magnetosome formation. We adjusted the ferric citrate concentration in the feed flask and controlled it at a constant level (~100 μmol l^-1^) throughout the course of experiment (Figure 3d). A total of 9.0 g of ferric citrate was fed to the 42 L fermentor containing 28.4 L of medium, and 8.1 g was assimilated into cells, for an uptake efficiency of ~90%. The theoretical Fe_3_O_4 _yield in magnetosomes in this case should be 65.76 mg l^-1 ^(no combined H_2_O) or 73.60 mg l^-1^(contains 10% of combined H_2_O); the practical magnetosome yield was 83.23 ± 5.36 mg l^-1 ^(dried using a vacuum freeze drier) or 75.96 ± 4.99 mg l^-1^(dried at 105°C for 24 hrs). These results also indicate that a large amount of the ferric iron taken up is associated with magnetosome formation after the DO became undetectable. Electron micrographs of cells in large scale culture in various phases show chain arrangements of magnetosomes after DO became undetectable for 1-2 hr (Figure 5), consistent with the findings of Staniland et al. [40].

## Discussion

Several groups have investigated magnetosome formation in large scale cultures of *M. magneticum *AMB-1 (including recombinant forms) and *M. gryphiswaldense *MSR-1 [34,35,41,42] and through improvement of culture conditions, the magnetosome yield has increased progressively from 4.7 mg l^-1 ^(or 2.4 mg l^-1^day^-1^) to 41.7 mg l^-1 ^(or 16.7 mg l^-1 ^day^-1^,). Control of dissolved oxygen (DO) in the medium within a low and narrow range (< 0.2 ppm [41], 0.25 mbar [36], 2~7 μmol l^-1 ^[40] (equivalent to 1.7~6.0 mbar; 1 bar = 10^5^ pa) is essential for magnetosome formation. Therefore, amplification for large-scale cultivation will require precise electrodes for measurement of DO. The oxygen electrodes presently used in large fermentors are not sufficiently sensitive for culture of magnetotactic bacteria. To resolve the paradoxical situation that the cell growth requires higher DO whereas magnetosome formation requires low DO below the detectable range of regular oxygen electrode, DO was controlled to optimal level using the change in cell growth rate [38]. In this study, DO was controlled at undetectable level for magnetosome formation whereas cell growth improvement has been further refined by adjusting stirring rate and air flow under chemostat culture conditions.

Low concentrations of nutrients in medium, special carbon source were the other key limiting factors that affect cell density of all magnetotactic bacterial cultures. Up to now, just only several organic acids were used as carbon source for cultivation of MS-1, AMB-1 and MSR-1, whereas NaHCO_3 _for MC-1 and MO-1. Our results show that it is important to keep the sodium lactate concentration low for rapid growth of MSR-1, and to maintain low DO for magnetosome formation in cells. Since controlling sodium lactate at a low level is difficult in sizeable scale-up, specific feeding strategies and feeding parameters needs to be adopted for auto-fermentors in the laboratory. However, this approach is challenging in large-scale industrial bacterial cultivation because of differences in the types of fermentors and the difficulty in regulating the carbon source required maintain cultures under such conditions. This problem was overcome in our study by using chemostat culture technology with pH-stat feeding and we achieved a high density of MSR-1 cells in a 42 L auto-fermentor and this fundamental research lays a basic foundation for the establishment of much larger scale production of mangetosome in fermentation industry.

More than 80% of ferric ion absorption rate occurred in the log phase of cell growth and this correlated with magnetosome formation after the DO became undetectable (Figure 4, 5b-e). These data suggest that Fe^3+ ^was likely transferred into cells as an electron acceptor for magnetosome biosynthesis to compensate oxygen insufficiency. It is well known that oxygen usually serves as a terminal electron acceptor to generate ATP for living organisms. In the couple 1/2 O_2_/H_2_O, which has a reduction potential (*E0'*) of +0.82 volts (V), H_2_O has a reduced tendency to donate electrons, but O_2 _has a high tendency to accept electrons. The reduction potential of the Fe^3+^/Fe^2+ ^couple is +0.2 V (pH 7) whereas +0.76 V (pH 2) [43]. Hence, under conditions where oxygen is absent, Fe^3+ ^can function as an electron acceptor. In previous studies with AMB-1, growth with nitrate has been reported to result in higher yields of magnetosomes [44] which was further increased by lowering the nitrate level in chemostat cultures by pH-stat feeding [45]. Similar to earlier results, our study showed that cells preferred to use NO_3_^-^/NO_2_^-^couple (+0.42 V) as an electron acceptor, than Fe^3+^/Fe^2+ ^couple, and resulting in magnetosomes yields decrease.

## Conclusions

Compared to previously reported methods, our culture technique with the MSR-1 strain significantly increased cell density, cell yield, and magnetosome yield in a shorter time window and thus reduced the cost of production. This offers two advantages that allow easy upscaling of the process for industrial fermentors: (i) the concentrations of carbon, nitrogen, and iron source in the medium can be auto-controlled at a constant level by pH-stat feeding, leading to ease of manipulation and eliminating the possibility of nutrient exhaustion during the culture process; (ii) mass production of magnetosomes by MSR-1 in a large-scale fermentor can be achieved by solely adjusting the stirring rate and airflow as observed in our DO data (Figure 3). DO is the major factor affecting growth rate in these culture systems and we were able to control this parameter without using highly sensitive DO electrodes as described in our earlier study [38]. The cell density and magnetosome yield reported here are the highest so far achieved with a magnetotactic bacteria. Refinement of this technique will enable further increase of cell density and magnetosome yield.

## Methods

### Bacterial strain

*Magnetospirillum gryphiswaldense *MSR-1 (DSM6361) was purchased from Deutsche Sammlung von Mikroorganismen und Zellkulturen GmbH.

### Medium preparation

Flask culture was carried out in Na-lactate medium as described previously [38]. All medium components except K_2_HPO_4 _were dissolved in 5.4 L or 27 L distilled water in a 7.5 L or 42 L fermentor, respectively, and then sterilized for 30 min at 121°C. K_2_HPO_4 _was dissolved in 200 ml or 2 L distilled water, and then sterilized separately for 30 min at 121°C. Sterilized K_2_HPO_4 _solution was pumped into the fermentor before inoculation.

### Preparation of seed culture

A single colony of MSR-1 from Na-lactate medium agar plates was transferred to a tube containing 10 ml Na-lactate medium and grown with 100 r min^-1 ^orbital shaking at 30°C for 24 hr. Ten ml of this culture was inoculated into 90 ml fresh Na-lactate medium in a 250 ml bottle and incubated under the same conditions. This was used as the initial seed culture. A volume of 900 ml fresh medium in 3000 ml shaking flasks was inoculated with 10% (vol/vol) of initial seed culture and grown under the same conditions. On larger scales, 10% (vol/vol) of seed culture was inoculated into the fermentor for subsequent experiments.

### Growth conditions

Temperature and pH were controlled at 30°C and 6.8 during cultivation. pH was adjusted by nutrient solutions (containing 4.2 g ferric citrate, 129 g sodium lactate, 52.6 g lactic acid, and 54.9 g NH_4_Cl per liter). Initial air flow and stirring rate were controlled at 0.5 l min^-1 ^and 200 r min^-1^, respectively, in the 7.5 L fermentor.

### Cell density and cell dry weight

Cell growth (optical density) was measured spectrophotometrically at a wavelength of 565 nm. One OD_565 nm _unit corresponds to 0.3 g l^-1 ^dry cell weight. Magnetosomes were collected and purified as described previously [33], dried using a vacuum freeze drier (Kinetics, EZ550Q) or at 105°C for 24 hr, and weighed.

### Iron concentration

Aliquots of 1.0 ml of batch culture were centrifuged at 7000 g for 1 min. The supernatant was used for ferric ion or sodium lactate estimation. Ferric ion concentration was determined as described previously [46] with modification as follows. To 100 μl of sample, 50 μl of 5% hydroxylamine hydrochloride, 1 ml 15% tartaric acid, 5 ml 0.25% 1,10-phenanthroline, and 10 ml 25% glacial sodium acetic acid were added. After 15 min, the absorbance of sample solutions was determined spectrophotometrically at 510 nm.

### Lactic acid concentration

The concentration of lactic acid in the supernatant was analyzed by high performance liquid chromatography (HPLC) (Waters 510 system, USA) with Aminex HPX-87 H Organic Acid Analysis Column (Bio-Rad, USA), using a Waters 2414 Refractive Index Detector. The column temperature was 65°C; detector temperature was 45°C. A solution of 5 mmol l^-1 ^H_2_SO_4 _was used as mobile phase at 0.6 ml min^-1 ^flow rate.

### Transmission electron microscopy

Cells in the pellets were rinsed three times, suspended in distilled water, adsorbed onto a 300-mesh carbon-coated copper grid, and viewed directly by transmission electron microscope (Philips Tecnai F 30) at an accelerating voltage of 300 kV for recording magnetosomes.

## Competing interests

The authors declare that they have no competing interests.

## Authors' contributions

LY and LRG initiated and coordinated the project. LY and JW performed the batch cultivation.LY and GFF were responsible for transmission electron microscopy.

LY and LJL provided critical discussion and full support for this project. All authors wrote the paper and approved the final version of the manuscript.

## References

[B1] Blakemore R Magnetotactic bacteria Science 1975 190 4212 377 379 10.1126/science.170679 170679

[B2] Frankel RB Bazylinski DA Johnson MS Taylor BL Magneto-aerotaxis in marine coccoid bacteria Biophys J 1997 73 2 994 1000 10.1016/S0006-3495(97)78132-3 9251816 PMC1180996

[B3] Schüler D Formation of magnetosomes in magnetotactic bacteria J Mol Microbiol Biotechnol 1999 1 1 79 86 10941788

[B4] Bazylinski DA Frankel RB Lovley DR Biologically controlled mineralization of magnetic iron minerals by magnetotactic baceria Environmental microbe-metal interactions ed 2000 Washington DC: ASM Press 109 149

[B5] Bazylinski DA Frankel RB Magnetosome formation in prokaryotes Nat Rev Microbiol 2004 2 3 217 230 10.1038/nrmicro842 15083157

[B6] Lefèvre CT Bernadac A Yu-Zhang K Pradel N Wu LF Isolation and characterization of a magnetotactic bacterial culture from the Mediterranean Sea Environ Microbiol 2009 11 7 1646 1657 10.1111/j.1462-2920.2009.01887.x 19220399

[B7] Gould JL Kirschvink JL Deffeyes KS Bees have magnetic remanence Science 1978 201 4360 1026 1028 10.1126/science.201.4360.1026 17743635

[B8] de Araujo FF Pires MA Frankel RB Bicudo CE Magnetite and magnetotaxis in algae Biophys J 1986 50 2 375 378 10.1016/S0006-3495(86)83471-3 19431684 PMC1329754

[B9] Walcott C Gould JL Kirschvink JL Pigeons have magnets Science 1979 205 4410 1027 1029 10.1126/science.472725 472725

[B10] Kirschvink JL Homing in on vertebrates Nature 1997 390 399 340

[B11] Dunn JR Fuller M Zoeger J Dobson J Heller F Hammann J Caine E Moskowitz BM Magnetic material in the human hippocampus Brain Res Bull 1995 36 2 149 153 10.1016/0361-9230(94)00182-Z 7895092

[B12] Jogler C Schüler D Genomics, genetics, and cell biology of magnetosome formation Annu Rev Microbiol 2009 63 501 521 10.1146/annurev.micro.62.081307.162908 19575557

[B13] Xie J Chen K Chen X Production, Modification and Bio-Applications of Magnetic Nanoparticles Gestated by Magnetotactic Bacteria Nano Res 2009 2 4 261 278 10.1007/s12274-009-9025-8 20631916 PMC2902887

[B14] Jogler C Kube M Schübbe S Ullrich S Teeling H Bazylinski DA Reinhardt R Schüler D Comparative analysis of magnetosome gene clusters in magnetotactic bacteria provides further evidence for horizontal gene transfer Environ Microbiol 2009 11 5 1267 1277 10.1111/j.1462-2920.2009.01854.x 19220405

[B15] Matsunaga T Okamura Y Fukuda Y Wahyudi AT Murase Y Takeyama H Complete genome sequence of the facultative anaerobic magnetotactic bacterium Magnetospirillum sp. strain AMB-1 DNA Res 2005 12 3 157 166 10.1093/dnares/dsi002 16303747

[B16] Pradel N Santini CL Bernadac A Fukumori Y Wu LF Biogenesis of actin-like bacterial cytoskeletal filaments destined for positioning prokaryotic magnetic organelles Proc Natl Acad Sci USA 2006 103 46 17485 17489 10.1073/pnas.0603760103 17085581 PMC1859955

[B17] Schüler D Genetics and cell biology of magnetosome formation in magnetotactic bacteria FEMS Microbiol Rev 2008 32 4 654 672 10.1111/j.1574-6976.2008.00116.x 18537832

[B18] Nakamura C Burgess JG Sode K Matsunaga T An iron-regulated gene, magA, encoding an iron transport protein of Magnetospirillum sp. strain AMB-1 J Biol Chem 1995 270 47 28392 28396 10.1074/jbc.270.47.28392 7499342

[B19] Komeili A Vali H Beveridge TJ Newman DK Magnetosome vesicles are present before magnetite formation, and MamA is required for their activation Proc Natl Acad Sci USA 2004 101 11 3839 3844 10.1073/pnas.0400391101 15004275 PMC374331

[B20] Komeili A Li Z Newman DK Jensen GJ Magnetosomes are cell membrane invaginations organized by the actin-like protein MamK Science 2006 311 5758 242 245 10.1126/science.1123231 16373532

[B21] Scheffel A Gärdes A Grünberg K Wanner G Schüler D The major magnetosome proteins MamGFDC are not essential for magnetite biomineralization in Magnetospirillum gryphiswaldense but regulate the size of magnetosome crystals J Bacteriol 2008 190 1 377 386 10.1128/JB.01371-07 17965152 PMC2223754

[B22] Schüler D Molecular analysis of a subcellular compartment: the magnetosome membrane in Magnetospirillum gryphiswaldense Arch Microbiol 2004 181 1 1 7 10.1007/s00203-003-0631-7 14668979

[B23] Matsunaga T Sakaguchi T Molecular mechanism of magnet formation in bacteria J Biosci Bioeng 2000 90 1 1 13 16232810 10.1016/s1389-1723(00)80001-8

[B24] Matsunaga T Kamiya S Use of magnetic particles isolated from magnetotactic bacteria for enzyme immobilization Applied Microbiology and Biotechnology 1987 26 328 332 10.1007/BF00256663

[B25] Nakamura N Matsunaga T Highly sensitive detection of allergen using bacterial magnetic particles Analytica Chimica Acta 1993 281 585 589 10.1016/0003-2670(93)85018-F

[B26] Matsunaga T Sato R Kamiya S Tanaka T Takeyama H Chemiluminescence enzyme immunoassay using ProteinA-bacterial magnetite complex Journal of Magnetism and Magnetic Materials 1999 194 1-3 126 131 10.1016/S0304-8853(98)00575-7

[B27] Lee JH Huh YM Jun YW Seo JW Jang JT Song HT Kim S Cho EJ Yoon HG Suh JS Artificially engineered magnetic nanoparticles for ultra-sensitive molecular imaging Nat Med 2007 13 1 95 99 10.1038/nm1467 17187073

[B28] Chertok B David AE Huang Y Yang VC Glioma selectivity of magnetically targeted nanoparticles: a role of abnormal tumor hydrodynamics J Control Release 2007 122 3 315 323 10.1016/j.jconrel.2007.05.030 17628157 PMC2094531

[B29] McAteer MA Sibson NR von Zur Muhlen C Schneider JE Lowe AS Warrick N Channon KM Anthony DC Choudhury RP In vivo magnetic resonance imaging of acute brain inflammation using microparticles of iron oxide Nat Med 2007 13 10 1253 1258 10.1038/nm1631 17891147 PMC2917758

[B30] Magnani M Galluzzi L Bruce IJ The use of magnetic nanoparticles in the development of new molecular detection systems J Nanosci Nanotechnol 2006 6 8 2302 2311 10.1166/jnn.2006.505 17037835

[B31] Barakat NS Magnetically modulated nanosystems: a unique drug-delivery platform Nanomedicine (Lond) 2009 4 7 799 812 10.2217/nnm.09.66 19839815

[B32] Sun JB Duan JH Dai SL Ren J Zhang YD Tian JS Li Y In vitro and in vivo antitumor effects of doxorubicin loaded with bacterial magnetosomes (DBMs) on H22 cells: the magnetic bio-nanoparticles as drug carriers Cancer Lett 2007 258 1 109 117 10.1016/j.canlet.2007.08.018 17920762

[B33] Xiang L Wei J Jianbo S Guili W Feng G Ying L Purified and sterilized magnetosomes from Magnetospirillum gryphiswaldense MSR-1 were not toxic to mouse fibroblasts in vitro Lett Appl Microbiol 2007 45 1 75 81 10.1111/j.1472-765X.2007.02143.x 17594464

[B34] Matsunaga T Kawasaki M Yu X Tsujimura N Nakamura N Chemiluminescence enzyme immunoassay using bacterial magnetic particles Anal Chem 1996 68 20 3551 3554 10.1021/ac9603690 8865763

[B35] Heyen U Schüler D Growth and magnetosome formation by microaerophilic Magnetospirillum strains in an oxygen-controlled fermentor Appl Microbiol Biotechnol 2003 61 5-6 536 544 12764570 10.1007/s00253-002-1219-x

[B36] Yang C Takeyama H Matsunaga T Iron feeding optimization and plasmid stability in production of recombinant bacterial magnetic particles by Magnetospirillum magneticum AMB-1 in fed-batch culture J Biosci Bioeng 2001 91 2 213 216 10.1263/jbb.91.213 16232977

[B37] Villaverde A Nanotechnology, bionanotechnology and microbial cell factories Microb Cell Fact 2010 9 53 10.1186/1475-2859-9-53 20602780 PMC2916890

[B38] Sun JB Zhao F Tang T Jiang W Tian JS Li Y Li JL High-yield growth and magnetosome formation by Magnetospirillum gryphiswaldense MSR-1 in an oxygen-controlled fermentor supplied solely with air Appl Microbiol Biotechnol 2008 79 3 389 397 10.1007/s00253-008-1453-y 18425510

[B39] Wolin EA Wolin MJ Wolfe RS Formation of Methane by Bacterial Extracts J Biol Chem 1963 238 2882 2886 14063318

[B40] Staniland S Ward B Harrison A van der Laan G Telling N Rapid magnetosome formation shown by real-time x-ray magnetic circular dichroism Proc Natl Acad Sci USA 2007 104 49 19524 19528 10.1073/pnas.0704879104 18032611 PMC2148322

[B41] Schüler D Baeuerlein E Dynamics of iron uptake and Fe3O4 biomineralization during aerobic and microaerobic growth of Magnetospirillum gryphiswaldense J Bacteriol 1998 180 1 159 162 9422606 10.1128/jb.180.1.159-162.1998 PMC106862

[B42] Yang C Takeyama H Tanaka T Matsunaga T Effects of growth medium composition, iron sources and atmospheric oxygen concentrations on production of luciferase-bacterial magnetic particle complex by a recombinant Magnetospirillum magneticum AMB-1 Enzyme Microb Technol 2001 29 1 13 19 10.1016/S0141-0229(01)00343-X 11427230

[B43] Madigan MT Martinko JM Parker J Brock Biology of Microorganisms 2003 Upper Saddle River: Pearson Education

[B44] Matsunaga T Tsujimura N Enhancement of magnetic particle production by nitrate and succinate fed-batch culture of *Magnetospirillum *sp. AMB-1 Biotechnology Techniques 1996 10 495 500 10.1007/BF00159513

[B45] Matsunaga T Togo H Kikuchi T Tanaka T Production of luciferase-magnetic particle complex by recombinant Magnetospirillum sp. AMB-1 Biotechnol Bioeng 2000 70 6 704 709 10.1002/1097-0290(20001220)70:6<704::AID-BIT14>3.0.CO;2-E 11064341

[B46] Tamura H Goto K Yotsuyanagi T Nagayama M Spectrophotometric determination of iron(II) with 1,10-phenanthroline in the presence of large amounts of iron(III) Talanta 1974 21 4 314 318 10.1016/0039-9140(74)80012-3 18961462

